# Social acceptance of alcohol use in Uganda

**DOI:** 10.1186/s12888-020-2471-2

**Published:** 2020-02-07

**Authors:** Joshua Ssebunnya, Caroline Kituyi, Justine Nabanoba, Juliet Nakku, Arvin Bhana, Fred Kigozi

**Affiliations:** 1 Butabika National Referral Mental Hospital, Kampala, Uganda; 2 Health Systems Research Unit, South Africa Medical Research Council and Centre for Rural Health, School of Nursing and Public Health, University of KwaZulu-Natal, Cape Town, South Africa

**Keywords:** Alcohol, PRIME, Kamuli, Alcohol use disorder, Acceptance

## Abstract

**Background:**

Alcohol use is part of many cultural, religious and social practices, and provides perceived pleasure to many users. In many societies, alcoholic beverages are a routine part of the social landscape for many in the population. Relatively low rates were reported for Alcohol Use Disorders (AUD) in a community-based survey and facility detection survey conducted in the study site contrary to findings in earlier formative studies where alcohol use was reported to be a major health problem. The aim of this study was to understand the reasons for under-reporting and the low detection rate for AUDs, exploring societal perceptions of alcohol use in the study district.

**Methods:**

The study was conducted in Kamuli District (implementation site for the PRIME project). Semi-structured interviews and focus group discussions were conducted with purposively selected participants that included local and religious leaders, lay people, health workers as well as heavy alcohol drinkers and their spouses. Interviews were tape-recorded and transcribed verbatim. The analysis followed four thematic areas, which include the extent and acceptability of alcohol use, patterns of alcohol use, perceived health problems associated with alcohol use and help-seeking behavior for persons with alcohol related problems.

**Results:**

The findings indicate that alcohol consumption in the study site was common and widely acceptable across all categories of people and only frowned upon if the person becomes a nuisance to others. These findings suggest that the health problems associated with alcohol use are overlooked except when they are life-threatening. Help-seeking for such problems was therefore reported to be relatively rare.

**Conclusion:**

Alcohol was readily available in the community and its consumption widely acceptable, with less social sanctions despite the legal restrictions to the minors. The social acceptance results in low recognition of alcohol use related health problems, consequently resulting in poor help-seeking behavior.

## Background

Alcohol use is part of many cultural, religious and social practices, and provides perceived pleasure to many users. It is an ancient custom in many communities and has never been an illegal act [1]. In many societies, alcoholic beverages are a routine part of the social landscape for many in the population, viewed as socially useful and necessary [1, 2]. Globally, alcohol consumption has increased in recent decades, most especially in low- and middle-income countries [3]. An estimated 43% of the population worldwide (15+ years) are current drinkers [2].

One quarter of all alcohol consumed worldwide is in the form of homemade, unrecorded alcohol – i.e. alcohol that is not accounted for in the national official statistics. In low income countries, this is as high as 40% [2]. Such alcohol is often illegally produced, and its consumption may be associated with an increased risk of harm because of the unknown and potentially dangerous impurities in these beverages [2, 4].

Cultural values have been reported to have a powerful influence over the use of alcohol throughout the world, and in particular, Africans are accustomed to the consumption of fermented beverages which tend to have less alcoholic content than distilled beverages. Alcohol has been part of the social and religious life of Africa since the third century and continues to be an integral part of ceremonies such as naming children, marriage, funerals, judicial processes and legal contracts. Traditionally, the consumption of alcoholic beverages was restricted to elders of the community and drinking was social rather than an individual activity [5]. However, although alcohol consumption is largely socially acceptable in many societies, it has substantial effects on the health and well-being of individuals and the community [6]. It is the world’s third largest risk factor for disease and disability; and the greatest risk factor in middle-income countries [4, 7]. Injury and death caused by alcohol consumption have socioeconomic impacts. By 2011, approximately 4.5% of the global burden of disease and injury was attributable to alcohol; and it was the third highest risk for disease and disability, after childhood underweight and unsafe sex [4]. Alcohol contributed 5.9% of all the deaths globally in 2014 as compared to 3.8% in 2004 and 3.2% in 2000 [4, 8].

The prevalence of alcohol consumption in Uganda has been reported to be high, with differences among men and women attributable to culture and gender-based distinctions between the roles, responsibilities and expectations of men and women [6]. According to the 2004 Global Status Report on alcohol, Uganda had the highest annual consumption of alcohol in the world, with 19.47 l of pure alcohol being consumed per capita among persons aged 15 years and above; in addition to an unrecorded consumption estimated at 10.7 l of pure alcohol annually per adult [9]. Although subsequent reports have indicated a reduction in consumption, the country is still among those with the highest annual per capita consumption in the African region [2], and has no national monitoring system in place.

In a community-based survey and facility detection survey conducted earlier in Kamuli (a rural district in Uganda), the proportion of men who screened positive for alcohol use disorders (AUD) was 4.1 and 5.8% respectively, despite earlier reports of wide spread alcohol use as one of the major health problems in the district in the formative studies [10, 11]. It is against this background that a parallel study was conducted specifically to understand the reasons for the reported low prevalence and low detection rate for AUDs, exploring the societal perceptions of alcohol use in the district, patterns of use as well as perceptions of the consequences on health and behavior. The study was conducted as part of a broader multi-centre study; the PRogramme for Improving Mental health care (PRIME), a research consortium that set out to generate evidence on the implementation and scaling up of treatment programmes for priority mental disorders in primary and maternal health care contexts in five low resource settings. These priority disorders included Alcohol Use Disorders (AUD) among others [12].

## Methods

### Setting

The study was conducted in Kamuli District (implementation site for the PRIME project), a predominantly rural district located in Eastern Uganda, 140 km away from the capital city. Administratively, the district is made up of 2 counties, 10 sub-counties, 79 parishes and 755 zones/villages. For health service delivery, it is divided into 2 health sub-districts, each having several health facilities at various levels, as clearly described in another publication [13]. At the time of the study (in the year 2016), the district had a total fertility rate of 6.8, above the national average of 5.8, and was estimated to have a population of 500,800 people; with males constituting 48.1% and females 51.9% [14]. The population was predominantly young, with an estimated 59% being children below 18 years of age [15]. As a rural district, the main economic activity is farming, dominated by sugarcane growing. The district is typically representative of the majority rural districts in Uganda in light of its socio-economic and health indicators.

### Participants

Data was collected from various categories of respondents selected purposively and drawn from 6 out of the 9 sub-counties making up the district. These included religious leaders, health workers, local leaders, cultural leaders as well as lay people including heavy alcohol drinkers (persons reported to consume large quantities of alcohol daily), their wives and children.

### Data collection

Data collection was done by conducting Key Informant Interviews as well as Focus Group Discussions (FGDs) with study participants. The FGDs had 8–9 participants; and the study altogether involved 50 participants. In total, 16 Key Informant Interviews (KIIs) and 4 FGDs were conducted, as summarized in the Table 1 below:

**Table 1:** Summary of the key informant interviews and FGDs conducted

Heavy drinkers	1 FGD
Non-alcohol drinkers (lay people)	1 FGD
Wives of heavy drinkers	1 FGD
Children of heavy drinkers (aged 18–25 years)	1 FGD
Health facility managers	4 KIIs
Community Health Workers	2 KIIs
Local leaders	3 KIIs
Religious leaders	4 KIIs
Cultural leaders	1 KII
Non-alcohol drinkers (lay people)	2 KIIs

Data collection was done using an interview guide covering 4 broad themes (extent of alcohol use and acceptability, patterns of alcohol use, perceived health problems associated with alcohol use and help-seeking behavior for persons with alcohol related problems). All interviews and FGDs were tape recorded and transcribed verbatim. Interviews with health facility managers were conducted in English, and the rest were all conducted in the local language (Luganda). Interviews conducted in the local language were translated to English at the time of transcription. The interviewing and transcription were done by two project staff (graduates of Community Psychology and Social Work), conversant with both languages. The analysis was done by a senior researcher (Clinical Psychologist).

### Data analysis

Data analysis used a framework analysis approach [16], with the help of a qualitative data analysis package (NVivo9). A coding framework was initially developed based on the four broad themes listed above. Sub-themes were consequently generated, and data coded accordingly in NVivo9.

## Result

The findings are presented under four themes, in line with the study objectives. These include the extent and acceptability of alcohol use in the district, patterns of alcohol use, perceptions of problems associated with alcohol use as well as the help-seeking behavior of persons with alcohol use related problems.

### Reasons for alcohol use and social acceptance

Production and consumption of alcohol was noted to be a common practice in the district. This was largely attributed to the fact that Kamuli is a sugarcane growing district, and sugarcane is used in the production of local brew in addition to sugar. Participants affirmed that many households in the district rely on alcohol production as their main economic activity; making alcohol readily available, accessible and affordable, with minimal restriction to the young people. *It was further reported that in some areas, regular heavy drinkers would at times go to the local breweries and were allowed to consume alcohol for free when they did not have money.* Crude spirit processed from sugarcane was reported to be the most popular alcoholic drink used by the majority of the people in this area. The same product was reported to be a major business commodity attracting buyers from outside the district. Other relatively improved alcoholic beverages such as bottled/canned beer were reported to be available but less affordable and therefore not a preferred choice. Mixing brands of alcohol was reported to be a common practice among some of the heavy drinkers to realize the effect of intoxication more rapidly.

“…*some alcoholics prefer mixing Beer, locally produced Waragi, Malwa, Mwenge-bigere, Avon, Pass-palm, Malasi and Kuber into one container so that they feel the real effect of alcohol. But for that one, if you’re not senior or have never taken this mixture, you can end up defecating in public*” *(Respondent 4, FGD1, Alcohol drinkers).*

Respondents identified several reasons for alcohol consumption most notable of which was relaxation, socialization and the urge to drive away shyness and boldly approach potential sexual partners.

*“… like me who has no strength when having sex with a woman, alcohol gives me appetite to have sex. It boosts my energy so that I fulfill my duty of satisfying a woman sexually and she feels happy.” (Respondent 3, FGD1; Alcohol drinkers.)*

Others drink as a way of coping with losses, problems and dealing with their frustrations and unhealthy domestic relationships.

“…*you realize that some of us have wives who are very quarrelsome. You never have peace when with her at home. So, I rather spend time in drinking places than being at home with a quarrelsome wife. I only go home to sleep, to avoid quarrelling or fighting with her. It is o.k. to abuse me when am drunk…because I will have no energy to respond” (respondent 4, FGD 1 Alcohol drinkers).*

Introducing children to alcohol at an early stage was noted to be a common practice in this community. Children from families involved in alcohol production or sale, as a source of income often get directly involved in production and sale of alcohol. It emerged that some parents administer alcohol to children as medicine, with a belief that it cures cough, flu, malaria and kills intestinal germs. It was further reported that some parents proudly introduce their children to alcohol, especially the boys and are happy to have their sons drink, just like them. Furthermore, some of the participating heavy drinkers rationalized their drinking by referring to the bible since Jesus converted water to wine as one of the miracles, while others contended that it is very okay to drink on earth since there will be no alcohol in Heaven. The religious leaders on the other hand expressed their disapproval of alcoholism. They argued that the bible denounces alcoholism, and most alcoholics are entrapped into the vice, behaving under the control of the devil.

Alcohol consumption was therefore reported to be common and generally acceptable, as society tends to be less concerned about one’s drinking habits if he/she does not inconvenience others. Social events such as weddings, parties and other traditional festivities tend to be associated with excessive alcohol consumption.

Despite being legally and socially acceptable, some of the participants, *especially health workers and leaders* expressed mixed feelings towards alcohol consumption, describing alcoholics as wasted and irresponsible people who should not be trusted or respected. However, some others, especially the heavy drinkers had more positive views of alcohol as a beverage that has existed since time *immemorial,* a source of livelihood for families, and an important item traditionally used in paying pride price in many cultures. The drinking culture was said to be deeply entrenched in the community to the extent that some heavy drinkers formed support groups to promote their interests as alcoholics.

Most respondents believed that alcohol has no substantial health benefits to the individuals who consume it, apart from the short-lived pleasure and opportunity to interact with fellow alcoholics. They observed that, the major beneficiaries of the alcohol industry are individuals involved in production and sale of alcohol (as it is a source of income to them) as well as government through taxation.

### Patterns of alcohol use

Alcohol consumption was reported to be common across all categories of people in the district, irrespective of sex and religion. Born-again Christians and Muslims were however reported to drink in secrecy because their religions denounce alcoholism. *Consumption among females was reported to be relatively lower*, except those who work in bars and elderly women, especially widows. On the other hand, males were noted to start drinking alcohol at an early age.

“…*since alcohol is readily available in the homes, children begin drinking as early as four years and by the time they reach their 18years, they are already experts. They said, a child of 14 years can even drink more than a mature person. Such young alcoholics consequently age so fast*”.

Heavy alcohol drinkers were reported to be very social as they often drink in groups, as couples, families or peers. They apparently rationalize the behavior with the belief that alcohol drunk in isolation is not tasty and therefore always look out for their mates to drink together. In this way many can also get free alcohol. Underage drinking was particularly cited as one of the very serious problems in the community, since many minors take on drinking at a very early stage, mixing various brands of alcohol; at times combined with other illicit drugs. While alcohol was reported to be readily available and accessible in the district, consumption was reported to be particularly high in the sugarcane growing areas, where brewing is a key economic activity. The harvesting seasons as well as festive seasons were specifically said to be the peak periods, characterized by very heavy alcohol consumption beginning early in the day until late.

### Perceived health related problems

Heavy alcohol consumption was reported to be associated with some health problems, including susceptibility to sexually transmitted infections. The participants affirmed that due to excitement and impaired judgment, heavy drinkers often get involved in risky behaviours under the influence of alcohol; including irresponsible sexual behaviours that lead them to contracting various sexually transmitted infections, including HIV. Unmarried elderly women involved in the production, sale and consumption of alcohol were reported to seduce boys, buying them alcohol in exchange for sex.

“…*those old women around 40 years, who have constructed houses for themselves have gone beyond as far alcohol consumption is concerned. They are the major producers of locally made alcohol and in the process, they seduce young boys by buying for them alcohol in order to have sex with them and some of these boys have been married off by these women*” (FGD 1, male alcoholics).

Some of the heavy drinkers admitted having had unprotected sex, at times with fellow alcoholics under the influence of alcohol and regretting it later.

*“…and where we are getting problems is that men give women…they buy alcohol for women and at the end of the day, you find three men using the same woman because all of them have bought for her.” (KII 02, Local leader).*

Alcohol was also reported to be associated with poverty as alcoholics spend a lot of money on alcohol, depleting their meager resources and yet they continue being unproductive since they spend most of their time either in bars or at home sleeping after heavy drinking sprees. This often results in failure to meet the family’s basic needs, including children’s school fees consequently leading to problems such as domestic violence and child neglect.

### Help-seeking behavior

Seeking help for alcohol related health problems was reported to be rare except in emergencies, when one is experiencing a life-threatening condition due to alcohol. In most cases, it will be the family members, relatives or concerned significant others who attempt to seek help for their relatives or devise means of assisting them to stop their alcoholic habits after realizing adverse health effects.

“…*you think a drunkard can seek help by himself? No. Even if he falls sick, he doesn’t mind and if a friend happens to visit him during that time of his sickness and offers him some money to buy the drugs, he will use that money to buy alcohol. It is either a family or concerned relatives who struggle to see that they look for all ways of stopping this person from drinking, having realized the negative impacts.”* (FGD 02, Lay people [non-alcoholics]).

Voluntary help-seeking among alcoholics was thus reported to be extremely rare. Attempts to seek help from health facilities were reported to be made usually after acute intoxication and loss of consciousness or after sustaining severe physical injuries. While there were some health facilities within the district known to offer some help to those who wish to quit, some of the participants didn’t believe that health facilities can offer any help to alcohol dependants to reduce or stop their drinking. Those abusing alcohol in particular did not see any reason for seeking help at health facilities. Furthermore, the few persons with alcohol use disorders who seek help at health facilities were said to often present other physical health problems which the health workers focus on, overlooking the former. This consequently results in low detection and low reporting of alcohol use disorders at health facilities.

There were also reports of a very crude means of offering help (by lay people) to those abusing alcohol during acute intoxication by administering human urine; and some were reported to have lost lives in the process. Interventions to help persons abusing alcohol were reported to be hampered by the reluctance of the local authorities to enforce corrective measures partly because some of them are also involved either in the production or sale of alcohol. The law that prohibits consumption and sale of alcohol to minors is thus not implemented in this setting. The enforcement is further hampered by the local political leaders’ fear of annoying their potential voters, yet they themselves also usually buy and give out alcohol when soliciting for votes.

*“…now you see these days politics is a determining point in everything. Sometimes we take some issues to the local leaders but since they don’t want to destroy their votes, sometimes they don’t respond. They say that when they talk to these people and force them to take at least limited alcohol, or stop them, they will not give them votes.”*

## Discussion

According to the study findings, alcohol consumption in the district is a common practice, in line with earlier studies that have reported alcohol consumption to be a generally accepted social habit, especially in the poor countries where the revenue earned from the trade in alcohol constitutes a large percentage of the national income [1]. Study participants further affirmed that undocumented production and consumption of alcohol was common in the district, especially home-made alcohol. This finding agrees with some earlier studies that have documented the impact of alcohol supply, citing access as one of the leading determinants of alcohol consumption [17]. The situation is further worsened by the lack of stringent legal restrictions and laxity of the local authorities as regards implementation of the alcohol regulatory policies, which gives leeway to the general population, including minors to consume alcohol as they wish. The findings thus affirm a strong culture of alcohol acceptance in Uganda, a situation worsened by lack of an alcohol control policy and poor enforcement of laws [18]. The findings further indicated that some people get into the practice (alcohol consumption) to fit in with others or acting just like them, illustrating the concepts of social acceptance and peer influence. It is important to note that the populace of study site is characterized by cultural diversities, with some of the people from a particular ethnic group known to have a deep relationship with heavy alcohol consumption. This is in line with some earlier studies that have reported that the level of alcohol consumption tends to vary from culture to culture; depending fundamentally upon societal definitions and reactions [19, 20].

Excessive consumption of alcohol has been identified as one of the major causes of poverty in Uganda, as it leads to declined productivity, increased expenditure and loss of assets, impaired judgment and vulnerability to disease thereby being a driver and maintainer of chronic poverty [21]. Kamuli district is within Busoga sub-region, which is ranked the third poorest sub-region in the country [14]. Participants reported that those abusing alcohol in the area spent more time in drinking and related activities and believed this to contribute to the rampant poverty in the district. This observation highlights the relationship between alcohol and poverty; emphasizing the complexity of alcohol as a source of income for the poor, source of revenue for the government but at the same time named as one of the leading causes of chronic poverty in the country [22]. Alcohol consumption was also cited in a related study that compared the causes of household poverty in Bushenyi and Kamuli districts [23].

Alcohol abuse was reported to be common across all categories of people, although men were reported to be more heavily involved in drinking. In some areas, consumption was reported to start in the early hours of the day and go on till late, rendering the heavy alcohol consumers unproductive, which contributes to the high poverty levels in the area as earlier observed. Females were reported to drink less, which is in line with other earlier studies that regarded drinking alcoholic beverages as more of a masculine adult activity, with traditionally low rates among women in Africa. There were however some reports of high alcohol consumption by females, especially among single and elderly women. It should be noted that while women in African societies were previously punished if they drank excessively, this is not the case anymore, with increased social tolerance towards female drinking behaviour, making it less stigmatized [1, 20]. This change has been further attributed to increased availability of alcohol and changes in the role of women in the society [7].

Only a few participants commented on the health effects of alcohol citing a few health problems, including the susceptibility to HIV infection due to irresponsible sexual behavior under the influence of alcohol. This is in line with various other studies that have identified women’s alcohol use as a risk factor for HIV infection to be one of the negative consequences of harmful alcohol use [24, 25].

Despite scientific evidence suggesting that alcohol is a major source of health and social problems, the earlier studies reported low prevalence and detection of alcohol use disorders at health facilities [10, 11] alluding to the fact that there is poor appreciation of the health problems associated with heavy alcohol consumption. This is further affirmed by the reported low help seeking behavior among persons with alcohol related problems. The poor help-seeking behavior could further be attributed to limited awareness of the availability of services in this resource constrained community. This should be an area of concern for the local authorities and health workers, given the fact that, the study district scores poorly on the national health and socio-economic indicators [14].

## Conclusion

Alcohol was noted to be readily available, and its consumption legally and socially acceptable in the district, with few social sanctions despite the legal restrictions. Poor appreciation of alcohol use health-related problems was noted to be common, which in turn influences help-seeking behavior. Perceptions of excessive alcohol use as being culturally normative contributed largely to low help-seeking behavior or even recognizing alcohol abuse. This helps to explain the relatively low rates of identification found in the surveys.

## Recommendations

*We conclude by making the following recommendations in light of the findings:*

1. *There is need for finalization and wider dissemination of the National Alcohol Control Policy to guide strategies for curbing alcohol abuse and the associated problems.*
2. *The political administration should enact and enforce more strict bylaws and regulations to curb the growing problem of alcoholism in the district. Similarly, the health department should undertake tailored interventions within the communities (including health facilities) to address the problem of alcoholism and the associated adverse consequences.*
3. *Underage consumption of alcohol must be more severely controlled by restricting alcohol sales to minors and enforcing underage drinking laws. In addition, community-based efforts, in tandem with school-based prevention programmes which reinforce the message of potential harm of early alcohol use should be promoted.*
4. *The health department should prioritize population-based strategies and interventions, including community-wide awareness programmes. The strategy should be a collaboration with affected communities, highlighting excessive drinking as a contributing factor to health risks and poverty.*
5. *Social media platforms should be utilized to link prevention programmes with specific target audiences so that access and usability can be maximized.*

### Limitations of the study

The major limitation of this study was the fact that some of the participants were already drunk at the time of the group discussions and the views they expressed may have been compromised.

One other limitation is the fact that the study involved fewer respondents, given the nature of the data collection methods. Furthermore, the study had to include some respondents who were directly involved in alcohol production and consumption potentially leading to bias in favour of alcohol use.

## Data Availability

The data that support the findings of this study are available from the University of Cape Town data repository. The data files can also be accessed from the authors upon reasonable request and with permission of Makerere University College of Health Sciences.

## Abbreviations

AUD: Alcohol Use Disorder
FGD: Focus Group Discussion
HIV: Human Immunodeficiency Virus
KII: Key Informant Interview
PRIME: PRogramme for Improving Mental health carE
